# Carboxylesterase Factors Influencing the Therapeutic Activity of Common Antiviral Medications Used for SARS-CoV-2 Infection

**DOI:** 10.3390/pharmaceutics17070832

**Published:** 2025-06-26

**Authors:** Yue Shen, William Eades, Linh Dinh, Bingfang Yan

**Affiliations:** Division of Pharmaceutical Sciences, James L. Winkle College of Pharmacy, University of Cincinnati, Cincinnati, OH 45229, USA; sheny6@mail.uc.edu (Y.S.); eadesws@mail.uc.edu (W.E.)

**Keywords:** carboxylesterases, molnupiravir, nirmatrelvir, remdesivir, COVID-19

## Abstract

Severe acute respiratory syndrome coronavirus 2 (SARS-CoV-2), the virus responsible for COVID-19, remains a major global health threat. The virus enters host cells by binding to the angiotensin-converting enzyme 2 (ACE2) receptor. Several small-molecule antiviral drugs, including molnupiravir, favipiravir, remdesivir, and nirmatrelvir have been shown to inhibit SARS-CoV-2 replication and are approved for treating SARS-CoV-2 infections. Nirmatrelvir inhibits the viral main protease (M^pro^), a key enzyme for processing polyproteins in viral replication. In contrast, molnupiravir, favipiravir, and remdesivir are prodrugs that target RNA-dependent RNA polymerase (RdRp), which is crucial for genome replication and subgenomic RNA production. However, undergoing extensive metabolism profoundly impacts their therapeutic effects. Carboxylesterases (CES) are a family of enzymes that play an essential role in the metabolism of many drugs, especially prodrugs that require activation through hydrolysis. Molnupiravir is activated by carboxylesterase-2 (CES2), while remdesivir is hydrolytically activated by CES1 but inhibits CES2. Nirmatrelvir and remdesivir are oxidized by the same cytochrome P450 (CYP) enzyme. Additionally, various transporters are involved in the uptake or efflux of these drugs and/or their metabolites. It is well established that drug-metabolizing enzymes and transporters are differentially expressed depending on the cell type, and these genes exhibit significant polymorphisms. In this review, we examine how CES-related cellular and genetic factors influence the therapeutic activities of these widely used COVID-19 medications. This article highlights implications for improving product design, targeted inhibition, and personalized medicine by exploring genetic variations and their impact on drug metabolism and efficacy.

## 1. Introduction

The COVID-19 pandemic, caused by the SARS-CoV-2 virus, has profoundly affected healthcare systems and communications worldwide. Since 2019, the illness has been widespread, causing severe respiratory complications and significant mortality across continents [1]. The unprecedented health emergency has triggered an exponential increase in research, aiming to understand and mitigate the virus’s impact. The advent of vaccines targeting the SARS-CoV-2 spike (S) protein was a major milestone in combating COVID-19. However, the rapid mutation rate of the S protein has necessitated ongoing updates to vaccine formulations, potentially on a seasonal basis, akin to influenza vaccines.

**Figure 1:**
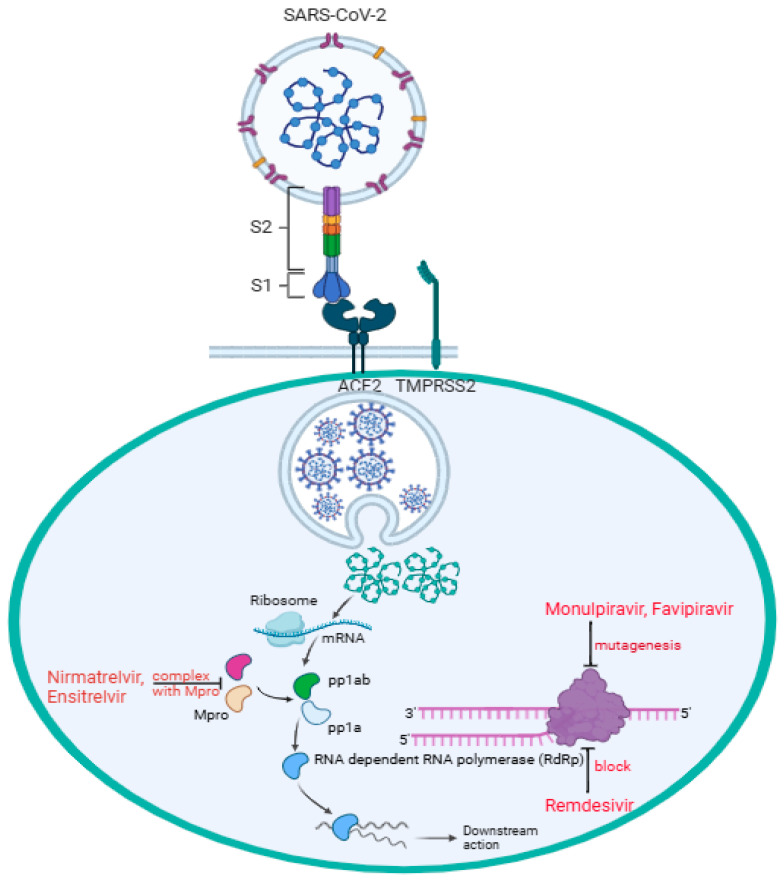
SARS-CoV-2 enters the target cell, particularly pneumocytes, through direct interaction between the cell surface ACE2 receptor and RBD B of the virus subunit S1, while the subunit S2 facilitates fusion between the host and the viral cell membranes. Followed by viral endocytosis, viral genomic mRNA is released into the host cell cytoplasm. Two-thirds of the mRNA, mainly at the 5′ end, encoded open reading frames (ORF1a and ORF1b), which are translated into two polypeptides, pp1a and pp1ab, and the remaining one-third of the mRNA, mainly at the 3′ end, serves as a template for transcription and replication. Nirmatrelvir and ensitrelvir form a complex with M^pro^, stopping pp1a and pp1ab from being processed into functional non-structural proteins (nsps) 4–16 for viral replication. Remdesivir blocks RdRp (nsp12) by mimicking adenosine (A) nucleoside incorporated into the RNA strand, preventing the virus from replication. Through RdRp, molnupiravir mimicking cytidine (C) or uridine (U) nucleosides introduces multiple mutations during replication, causing lethal mutagenesis, and effectively stopping the downstream action. Through RdRp, favipiravir mimicking guanine (G) gets incorporated into RNA strands, inducing mutations, thus causing lethal mutagenesis.

The binding of SARS-CoV-2 to the angiotensin-converting enzyme 2 (ACE2) receptor is a critical step in viral entry into the cells [2]. In Figure 1, the virus S1 subunit is binding with the host ACE2 receptor directly at the receptor-biding domain (RBD) B with the presence of the transmembrane protease, serine 2 (TMPRSS2), expressed on the surface of the respiratory epithelial cells. TMPRSS2 facilitates the activation of the S protein of SARS-CoV-2 infection, allowing the virus to fuse with the cell membrane and initiate infection [2,3]. Mutations in RBD B can enhance binding affinity and increase infectivity; thus, variants like alpha, beta, delta, and omicron have notable RBD mutations affecting transmissibility and immune escape [2,4]. In addition, the polybasic furin cleavage site (FCS) located between S1 and S2 subunits increases SARS-CoV-2 transmissibility [5,6,7]. Some studies suggest that the presence of O-linked glycans, sugar molecules attached to the S protein via oxygen-linked glycosylation, may play a role in immune evasion and host interactions [8,9]. This reflects the challenge of establishing long-lasting immunization strategies against the evolving virus. In parallel, antiviral treatments such as remdesivir, molnupiravir, and nirmatrevir have played an essential role as therapeutic interventions in managing the infectious disease by targeting viral replication. Remdesivir, molnupiravir, and nirmatrelvir have been authorized for use by the FDA and offer a treatment option that does not rely on specific viral strains. In this review, we examine how enzymatic, cellular, and genetic factors influence the therapeutic activities of remdesivir, molnupiravir, and nirmatrelvir against COVID-19.

## 2. Background

### 2.1. Antiviral Drugs for the Treatment of COVID-19

Table 1 lists antiviral drugs, including products that are required to be metabolized into their active forms. Most antiviral drugs are prodrugs since their parent drugs may not be as well absorbed, distributed, and activated at the site of infection. Another reason that prodrugs are used is that some active drugs degrade too quickly before reaching their target site of action. Moreover, some drugs are formulated to be selectively activated only within specific infected or targeted cells. Targeted activation by enzymes that are overexpressed in diseased or cancerous cells enables site-specific drug release, thus, reducing side effects [10]. Remdesivir was the first antiviral approved for COVID-19, exhibiting efficacy against SARS-CoV-2 by inhibiting the viral RNA-dependent RNA polymerase (RdRp). After being administered intravenously, remdesivir gets converted into an active nucleotide analog. Remdesivir incorporates into viral RNA chains, resulting in premature termination of viral replication [10,11,12]. Molnupiravir indirectly targets RdRp of SARS-CoV-2 by introducing a mutation into the viral genome through its active form NHC (β-D-*N*^4^-hydroxycytidine)-triphosphate, leading to lethal mutagenesis [13,14,15]. Favipiravir is a prodrug of guanosine (G) analog that inhibits RdRp and induces viral mutations, causing mutagenesis. However, favipiravir is not FDA-approved due to concerns about its inconsistent results against COVID-19 in clinical trials [14,15,16,17]. Molnupiravir, favipiravir, and remdesivir are prodrugs targeting RdRp; these nucleoside analogs typically exhibit poor cellular permeability. Phosphoramidate prodrug forms can significantly enhance their cellular uptake and metabolic activation. However, remdesivir resistance has been linked to mutations in the RdRp gene, such as E802D, which impede the drug incorporation into viral RNA [18]. Although molnupiravir has been reported to have a high barrier to drug resistance [19], mutations in RdRp, particularly within the nsp12 region, have been identified and may affect molnupiravir binding [20].

Nirmatrelvir, an active antiviral compound that inhibits proteases, on the other hand, bypasses the need for metabolic activation. Nirmatrelvir is often co-administered with ritonavir, a pharmacokinetic enhancer that can inhibit cytochrome P450 (CYP) metabolism, thereby increasing nirmatrelvir’s plasma concentration to block the viral main protease [M^pro^, also known as 3-chymotrypsin-like proteases (3CL^pro^)]. By inhibiting M^pro^, nirmatrelvir prevents the cleavage of polyproteins necessary for viral replication [21]. Recent results from the Phase 3 SCORPIO-PEP trial highlighted a breakthrough in COVID-19 treatment and prevention: Ensitrevil, a M^pro^ inhibitor, became the first and only oral antiviral to demonstrate significant efficacy as post-exposure prophylaxis against SARS-CoV-2 [22]. Figure 1 shows the mechanisms of remdesivir, molnupiravir, and nirmatrevil targeting the SARS-CoV-2 virus. An active drug, like nirmatrelvir, directly works against the virus or modulates the immune system without requiring metabolic activation. Resistance-conferring mutations, such as P132H and M49L occurred in the SARS-CoV-2 M^pro^ nsp5 domain, have been observed in vitro and in some circulating strains [23,24]. Nevertheless, nirmatrelvir may be more beneficial for COVID-19 patients who have metabolic issues such as liver failure.

While developing antiviral drugs represents a significant scientific breakthrough for treating COVID-19, emerging research suggests their effectiveness may be variable, especially for prodrugs. For instance, in studies conducted on SARS-CoV-2 infected Vero E6 cells, the EC_50_ values of remdesivir were reported at different values of 0.77 μM by Wang et al. [25,26,27], 1.65 µM by Pruijssers et al. [25,26] and ranging from 0.66 to 5.63 [28]. Experiments on cell lines have revealed that the same treatment produces varying quantities of active products. These observations highlight the need for a deeper understanding of the mechanisms governing drug action both within cells and systemically.

**Table 1:** List of antiviral drugs for the treatment of COVID-19.

Drug Name	Administration Route	Prodrug Activation	Mechanism of Action	Note
Remdesivir (Veklury^®^)	Intravenously infusion	remdesivir triphosphate (RTP)	RdRp inhibitor	
Nirmatrelvir/Ritonavir (Paxlovid™) ^1^	Oral	Nirmatrelvir is not a prodrug	Protease inhibitor	Emergency Use Authorization (EUA) in Dec. 2021, later with full approval
Molnupiravir (LAGEVRIO™)	Oral	NHC triphosphate	RdRp inhibitor	EUA ^2^
Favifpiravir	Oral	favipiravir ribofuranosyl-5′-triphostphate	RdRp inhibitor	
Ensitrelvir	Oral	Ensitrelvir is not a prodrug	Protease inhibitor	

^1^ In Paxlovid™, ritonavir is used not for its antiviral activity, but to increase the concentration of nirmatrelvir by inhibiting CYP3A4, thus, prolonging nirmatrelvir’s half-life [29]. ^2^ LAGEVRIO™ (molnupiravir) is prescribed only if Paxlovid™ and remdesivir are not options for mild to moderate COVID-19 in patients at risk, under FDA EUA.

### 2.2. Enzymes Involved in Antiviral Drug Activation and Metabolism: Carboxylesterases

Carboxylesterases (CES) belong to an esterase enzyme family that hydrolyzes esters, amides, thioesters, and carbamates into their corresponding alcohol and carboxylic acid. CES is widely distributed throughout the body [30]. Among the CES family, CES1 and CES2 are primarily involved in the activation and metabolism of prodrugs by hydrolyzing ester bonds in prodrugs, converting them into their active metabolites, which can exert therapeutic effects [30,31]. For example, remdesivir is metabolized in the liver by CES1, converting it to remdesivir active metabolites, which are then phosphorylated into RTP inside the cells, inhibiting viral replication [10,32,33]. In contrast, molnupiravir is hydrolytically activated by CES2 [34]. Interestingly, our previous study demonstrated that remdesivir at nanomolar concentrations could inhibit CES2 through covalent modifications, while no inhibition was detected on CES1, indicating the high specificity of the inhibition [35].

Additionally, given the fact that interspecies variability in carboxylesterase activity can significantly impact prodrug activation, and some animal studies can provide valuable translational insights [36,37], this review focuses on COVID-19 treatment in human subjects to ensure relevance to human pharmacokinetics and metabolism. Moreover, a respiratory syncytial virus inhibitor (e.g., ST-2) exhibited greater metabolic stability in human blood compared to mouse and rat blood due to lower carboxylesterase activity in humans, which enhances hydrolysis resistance, thus, highlighting improved pharmacological response in humans, especially in viral infections in the lung [36].

Systemic inflammation and cytokine storms during viral infections can cause hepatic drug-metabolizing enzyme suppression [38]. Thus, we believe that CES is downregulated and CES function could be transiently reduced during SARS-CoV-2 infection. Although direct evidence for CES downregulation in the context of COVID-19 is limited, the link between the viral infection and CES dysregulation can be supported by transcriptomic analyses of liver biopsies from COVID-19 patients [39] and proteomic studies with evidence of dysregulated hepatic protein networks in COVID-19 [40,41].

## 3. Carboxylesterase 1 (CES1)

### 3.1. CES1 Expression and Substrate Specificity

Although CES1 and CES2 are two dominant enzymes involved in drug metabolism, they exhibit distinct distributions and substrate types [25,26,27,28,29,30]. CES1 is mainly expressed in the liver and gall bladder, as well as in the lungs and subsets of cells in the gastrointestinal (GI) tract. CES1 (65.52 kDa) is the most abundant enzyme in the liver and plays an important role in the metabolism of esters, thioesters, and amides. CES1 is encoded by the CES1 gene, located on chromosome 16q12.2.

### 3.2. CES1 Pharmacogenetic Variability

Several CES1 variants have been shown to influence the efficacy of medications and clinical outcomes, highlighting the importance of individual genetic variation in drug metabolism (Table 2). The G143E CES1 variant has been observed in various clinical populations, with a frequency ranging from 2.5% to 5.8% depending on the cohort [42,43,44,45,46,47,48]. The G143E variant can affect the functionality of CES1, thus, affecting the efficacy and safety of drugs that rely on CES1 for activation/inactivation, including remdesivir. Interethnic variability in CES1 polymorphisms has significant implications in the context of a global pandemic like COVID-19 [42]. Population-based frequency data of G143E and rs2244613 is shown in Table 2. The prevalence of rs2244613 [47,49,50,51,52], as well as other genetic polymorphisms of the CES1 gene (Table 2), suggests pharmacogenomic factors related to dosing and drug responses could be significant for drugs like remdesivir. It is important to note that CES1 exhibits various genetic variability, and the variants that have been found to significantly impact CES1 enzyme function have been well-documented. Identifying the variants and their prevalence can help with dosing adjustments and tailoring drug therapy based on pharmacogenomic data, as well as providing valuable insights into the genetic variability of response to treatment within a certain population. When a drug is administered as a prodrug to the body, it is converted to its active form by CES and simultaneously transported and metabolized by P-glycoprotein (P-gp) and CYP enzymes in the liver and intestines. Therefore, CES, P-gp, and CYP genetic variants and their connections can potentially affect the metabolism and the plasma level of remdesivir. Meanwhile, CYP genetic variations can affect the metabolism of nirmatrevir and remdesivir, as both drugs undergo oxidation by CYP [53]. In clinical settings, the choice between remdesivir and Paxlovid™ should depend a lot on drug metabolism, whether the patient with COVID-19 infection has CES or CYP genetic variations, and how much CYP involvement determines how well the patient responds to the treatment.

## 4. Carboxylesterase 2 (CES2)

### 4.1. CES2 Expression and Substrate Specificity

For patients with CYP genetic variations, remdesivir is generally a safer choice since its activation depends on CES rather than CYP, but for patients who receive remdesivir as a COVID-19 treatment, there is a risk of drug-drug interactions with other medications metabolized by CES2 since remdesivir has been shown to inhibit CES2 [35]. Due to its high potency and irreversible inhibition, caution is advised when using remdesivir alongside medications that are hydrolyzed by CES2 such as molnupiravir [34], gemcitabine prodrugs [59], irinotecan [59,60], clopidogrel [59,61], vicagrel [61], orlistat [62], and even lipid-based drug/drug delivery systems since CES2 is known for being responsible for lipid metabolism in the intestines. CES2, which is found in the liver and intestines, hydrolyzes esters that contain a large alcohol group and a small acyl group, while CES1 hydrolyzes esters that contain a small alcohol group and a large acyl group. The crystal structure of mouse CES2 was reported to have structural parallels with human CES1 in substrate regulation and release [37].

### 4.2. CES2 Pharmacogenetic Variability

Like CES1, CES2 also plays a key role in hydrolyzing drugs, which can affect how certain drugs are activated or deactivated in the body, thus, genetic variations in CES2 can affect how quickly a drug is metabolized, how much active drug is available in the system, and the duration of its effect (Table 3). Interestingly, CES2 is more polymorphic across Asian populations, as exemplified by studies in Japanese individuals [42,63,64]; however, further research is needed to definitively confirm a strong association between rs2241409 and reduced CES2 activity, specifically in Asian populations. In general, CES1 is considered more well-studied than CES2; therefore, the clinical relevance of CES1 and the impact of CES1 variants on prodrug activation, especially G143E, has been well demonstrated. Several assertions regarding CES variants are based on isolated in vitro studies. This review compiles available data on CES variants affecting prodrug activation and takes into consideration findings from various studies, including in vitro studies. Some are isolated in vitro studies without further evidence for consistency or reproducibility across diverse experimental models. While we aim to be comprehensive, it is important to differentiate high-confidence and preliminary evidence for accurately interpreting the implications.

## 5. Drug Metabolism on the Therapeutic Activity of SARS-CoV-2 Antiviral Drugs

**Figure 2:**
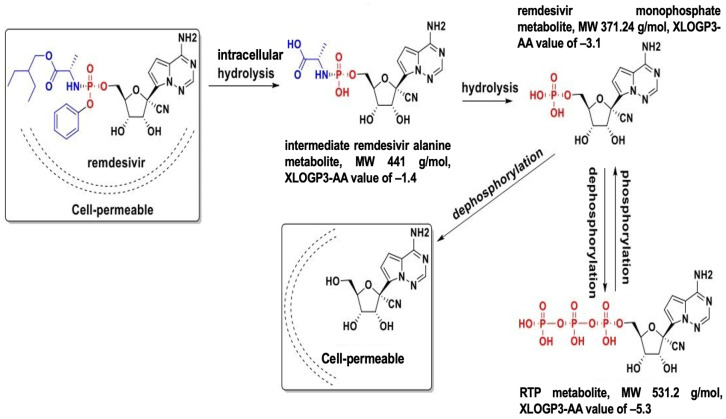
Step-by-step bioactivation of remdesivir.

**Figure 3:**
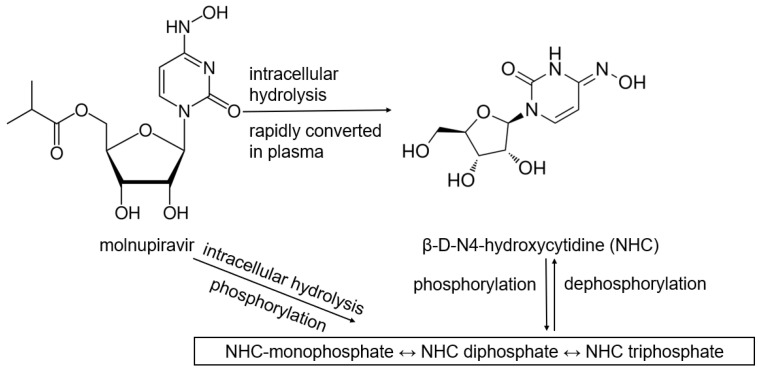
Bioactivation scheme of molnupiravir.

It is worth noting that all the prodrugs that are found to have clinical relevance with CES genetic polymorphism were reported to require only a one-step hydrolysis to become their active metabolite. To the best of our knowledge, no known variant has been linked to multi-step activation. There is a lack of clinical trials for prodrugs that consider multi-step activation, where hydrolysis is only the first step. Plenty of drugs require hydrolysis followed by phosphorylation or oxidation. Some CES1 substrates are being exemplified, for example, tenofovir alafenamide (TAF), an oral antiviral drug, undergoes hydrolysis to become tenofovir (TFV), and then TFV is phosphorylated twice to become TFV-diphosphate with antiviral activity [70]. Hydrolysis is the initial phase of TAF activation, thus, modifying the drug’s hydrophilicity and subsequent path through the body. The metabolic activation of remdesivir and molnupiravir is well characterized (Figure 2 and Figure 3) using standard in vitro models such as primary human hepatocytes or intestinal enterocytes. However, conventional hepatic models may overestimate prodrug activation capacity and limit the understanding of drug bioactivation in critical SARS-CoV-2 infection sites such as the lung and the GI tract. For instance, remdesivir requires CES1 activation, yet the presence of CES1 in the lung is minimal compared to ACE2 and TMPRSS2. A549 and Calu-3 cells have both CES1 and CES2 expressions, but available reports on their use in SARS-CoV-2 studies focus on their expression of ACE2 and TMPRSS2. Similarly, organoids and 3D cultures often prioritize ACE2 and TMPRSS2 expressions, but do not consistently measure CES activity.

### 5.1. Remdesivir

As mentioned above, remdesivir requires hydrolysis followed by phosphorylation to become its active form. The hydrolysis of remdesivir includes two steps: firstly, remdesivir is hydrolyzed into its alanine metabolite (GS-704427) by CES1 and cathepsin A, depending on the target organ; secondly, GS-704427 is transformed into GS-441524 mono- and triphosphate by phosphoamindase (Figure 2) [32,33,35,71,72]. Moreover, remdesivir has been reported to be a substrate of CYP2B6, CYP2C8, CYP2D6, and CYP3A4 [35,53,73,74]. The CYP oxidation of remdesivir occurs at the para position of the phenyl ring. The involvement of CES1, capthesin A, and phosphoamindase in the metabolism of remdesivir oxidative metabolite remains to be determined. In addition, G143E has been proven to be a loss-of-function variant of CES1 in remdesivir hydrolysis. Consequently, the impact of G143E and possibly other genetic polymorphisms on drug transmembrane transport and drug elimination must be considered.

Remdesivir shows higher lipophilicity than its metabolites with a higher logP, pointing to passive infusion favoring remdesivir over remdesivir metabolites. GS-441524, the nucleoside core, with a half-life of 26.6 h and steady blood concentration at 100 nM after administration of 0–24 h, is the predominant remdesivir derivative identified in the urine [75,76,77]. Remdesivir monophosphate and RTP undergo phosphorylation and dephosphorylation (Figure 2). RTP (GS-441524-triphosphate) with a 5.3 XlogP3-AA value is highly hydrophilic and has an anionic charge on the molecule, which makes it remain in the cellular compartment for a long period. It is reported that intracellular RTP level detected compared to remdesivir, after 24 h, continuous incubation with remdesivir resulted in 7.8-, 23-, and 23-fold higher than continuous incubation with RTP in Hep-2, PC-3, and primary hepatocytes [78]. In another study with peripheral blood mononuclear cells (PBMC), despite the intracellular concentration of RTP being approximately 220- to 370-fold above the in vitro EC_50_ value against SARS-CoV-2 in PBMC cells, remdesivir is undetectable in plasma [79], suggesting that the contribution of different kinases to remdesivir activation needs to be determined.

Although lung epithelial cells primarily express ACE2 and TMPRSS2, making them more susceptible to SARS-CoV-2 infection, it is reported that ACE2 and TMPRSS2 are highly expressed in the digestive tract; thus, COVID infection has been accompanied by GI symptoms such as GI bleeding, diarrhea, epigastric discomfort, etc., in several clinical reports. Since remdesivir and molnupiravir’s activation requires hydrolysis followed by multi-step phosphorylation, the manifestation of various intracellular enzymes will have a substantial effect on the drug’s efficacy.

In our previous studies, we highlighted remdesivir’s high specificity CES2 inhibition [35], but it is essential to consider this within the broader context of competitive versus mechanism-based inhibition. Competitive inhibition is reversible and concentration-dependent, where the drug occupies the active site of CES2. In contrast, irreversible enzyme inactivation through covalent modification following metabolic activation of the drug results in time-dependent loss of function of CES2. This irreversible mechanism-based inhibition can lead to prolonged drug-drug interactions. We conclude that remdesivir selectively targets the structure or catalytic features of CES2 via mechanism-based inhibition at nanomolar concentrations and that CES1, the more dominant esterase in the liver, remains unaffected. This effect can lead to altered metabolism of co-administered CES2 substrates, increasing the risk of drug-drug interactions. Our findings underscore the need for careful evaluation of CES2-related metabolic pathways during remdesivir therapy, especially in a COVID-19 setting.

Not only remdesivir metabolism is considered to be heavily involved with hydrolases and not CYP enzymes, but remdesivir is also a substrate of several transporters including bile salt export pump (BSEP) encoded by the ABCB11 gene, multidrug and toxin extrusion (MATE) 1 protein encoded by the solute carrier (SLC) SLC47A1 gene, multidrug resistance-associated (MRP) 4 protein encoded by the ABCC4 gene, sodium taurocholate co-transporting polypeptide (NTCP) encoded by the SLC10A1 gene, organic anion transporting polypeptide (OATP) 1B1 and OATP 1B3 encoded by the SLCO1B1 and SLCO1B3 genes, and organic cation transporter (OCT) 1 encoded by the SLC22A1 gene, especially equilibrative nucleoside transporter (ENT) 1 and ENT 2 [74,80]. Remdesivir has a high affinity with ENT1 and ENT2 and potentially inhibits ENT-mediated uridine uptake (IC_50_ at 38 ± 2 μM for ENT1 and 73 ± 14 μM for ENT2) [80]. Moreover, another study conducted with Madin-Darby canine kidney (MDCK) cells confirmed that GS-441524 is a substrate of concentrative nucleoside transporter (CNT) 3 and ENT1, showing uptake increases 81.2–164-fold and 3.38–7.45-fold in CNT3- and ENT1-expressing cells, respectively [81]. Interestingly, knock-out of SLC29A3 encoded ENT3 in the Huh7 cell line mitigates remdesivir toxicity without decreasing its antiviral activity [82]. While both remdesivir and GS-441524 can cross cell membranes, due to GS-441524’s hydrophilic nature, it likely relies on the specialized membrane molecules, the CNT family, and the family of ENTs consisting of ENT1–4 transporters, such as ENT1, ENT2, and CNT1, CNT2, for cellular entry. The co-expression patterns of CNT and CES across tissues can modulate local drug concentrations. For example, high CES1 expression in the liver alongside abundant CNT/ENT transporters may favor efficient activation and uptake of remdesivir, whereas low co-expression in the lung or GI tract might limit local bioactivation, thereby affecting antiviral potency at primary infection sites.

It is worth noting that the blood concentration of remdesivir active metabolite is kept at around 100 ng/mL after the first day of administration, while the blood concentration of remdesivir reaches its highest at 3170 ng/mL and is kept for 1.5 h higher than 100 ng/mL. It can be explained by the passive infusion of remdesivir dominating the early stage after administration. Thus, the presence of CES1 and/or cathepsin A-infected cells plays an important role in the achievement of therapeutic effects.

### 5.2. Molnupiravir

Molnupiravir (EIDD-2801) is an orally administered prodrug of the ribonucleoside analog NHC, also known as EIDD-1931, which undergoes intracellular phosphorylation to its active triphosphate form [34]. Upon administration, molnupiravir is efficiently hydrolyzed by CES2. This rapid conversion explains the low plasma concentrations of molnupiravir, especially at single doses of ≤800 mg, where molnupiravir levels were generally below the limit of quantification. NHC quickly presents in plasma following molnupiravir administration, peaking at about 1–1.75 h, and its half-life is approximately 1 h. However, at higher doses (1200 and 1600 mg), molnupiravir could be detected at select time points (0.25–1.5 h postdose), with peak concentrations reaching up to 13.2 ng/mL in 0.25–0.75 h. At a 1600 mg dose, NHC reaches a maximum plasma concentration of 6350 ng/mL. Minimal urinary excretion of molnupiravir was observed, accounting for only approximately 0.002% of the administered dose, indicating that renal clearance of the intact prodrug is negligible. Renal elimination of NHC is dose-dependent, with 0.82% of the 50 mg dose and up to 6.70% of the 1600 mg dose being recovered in urine over 24 h post-dose, suggesting a non-linear relationship between dose and urinary recovery [83]. These pharmacokinetic characteristics highlight molnupiravir’s efficient systemic delivery of its active metabolite through first-pass hydrolysis. Molnupiravir was undetectable in lung tissues, both pre- and post-infection, due to its rapid conversion to NHC. Even so, NHC concentrations in lung tissue were detectable, with levels ranging from 15 to 19 nmol/g, confirming its tissue penetration and potential for respiratory antiviral activity [84].

Molnupiravir is enzymatically hydrolyzed by CES2 in the intestinal tract, converting into its active form, NHC. The protein level of CES2 is notably high in various intestinal cells, including enterocytes, endocrine, and goblet cells, highlighting its key role in molnupiravir activation during oral absorption [85,86,87,88]. Thus, molnupiravir is specifically designed to be given orally due to its stability in the stomach acid and its activation in the gut by CES2. In theory, remdesivir could be chemically modified with improved properties targeting the CES2 activation pathway to create an oral remdesivir version [89]. Prodrugs that are CES2 substrates may offer improved oral delivery options and patient compliance, especially in mild or outpatient cases.

The bioactivation scheme of molnupiravir involves a multi-step metabolic process that converts EIDD-2801 to EIDD-1931 and NHC-triphosphate, which is the active metabolite that inhibits viral RNA replication (Figure 3). Molnupiravir, an isopropyl ester prodrug of NHC, is hydrolyzed to NHC rapidly in plasma and tissues. NHC permeates through the cell membrane, forming NHC-mono-, di-, and triphosphate through phosphorylation by the cell kinase enzymes in the intracellular space. Cells naturally maintain a balance between phosphorylation and dephosphorylation; therefore, dephosphorylation can occur, thus, reversing the activation pathway and competing with incorporation by the viral polymerase. In the case of molnupiravir, excess nucleotidases and phosphatases can cause excess dephosphorylation, resulting in the deactivation of molnupiravir.

In terms of transporter interactions, molnupiravir itself is a weak substrate of CNT1 and not a substrate of CNT2, CNT3, ENT1, or ENT2, limiting its transporter-mediated uptake [90]. For molnupiravir to be efficacious in the cells, transporters like ENTs and CNTs are essential for facilitating the intracellular uptake of its active form, NHC, and molnupiravir intermediate metabolite. This step is crucial as molnupiravir disappears fast post-dosage, while NHC is the predominant form in the bloodstream. In vitro studies demonstrated that NHC is a moderate inhibitor of ENTs, particularly ENT1 (IC_50_ = 259 µM) and ENT2 (IC_50_ = 467 µM), and exhibits greater potency in this regard than molnupiravir (ENT1 IC_50_ = 701 µM, ENT2 IC_50_ = 851 µM). Furthermore, NHC shows strong inhibition towards CNT1, with an IC_50_ of 18.77 µM. NHC also inhibits CNT2-mediated uridine uptake (IC_50_ = 10.17 µM), achieving up to 80% inhibition at 200 µM. In contrast, molnupiravir exhibited much weaker interaction with CNT1 (IC_50_ = 84.89 µM) and minimal inhibition of CNT2 [91]. The variability suggests that intracellular accumulation and tissue-specific distribution of NHC could be influenced by both competitive transporter inhibition and intracellular nucleoside levels. Interestingly, the NHC cytotoxicity profile in HT-29 cells was unaffected by ENT3 knockout, indicating that toxicity may not be mediated through mitochondrial polymerase or ENT3-associated pathways [82]. Overall, NHC is a substrate for CNT1, CNT2, CNT3, and ENT2, and potentially for ENT1, as suggested by a modest (1.8-fold) increase in uptake in ENT1-transfected MDCK II cells. However, definitive ENT1-mediated transport of NHC could not be confirmed due to the presence of S-(4-Nitrobenzyl)-6-thioinosine, a known ENT inhibitor, which blocked endogenous uptake in control cells [91]. These findings underscore the complexity of NHC transport and highlight that multiple nucleoside transporters may contribute to its cellular uptake and distribution. Together, these pharmacokinetic and transporter interaction data provide important insights into the absorption, distribution, and cellular entry mechanisms of molnupiravir and its active metabolite, supporting its development as an antiviral agent targeting respiratory viral infections like COVID-19.

### 5.3. Nirmaltrevil

Given the mentioned variability in CES expressions across individuals and tissues, CES-dependent prodrugs such as remdesivir and molnupiravir may exhibit greater interindividual variability in drug activation and therapeutic response for COVID-19 treatment. However, in general, for patients with CES genetic variations, nirmatrelvir can be considered a better choice since its activation depends on CYP, particularly CYP3A4, rather than CES. With ritonavir, renal excretion becomes the main elimination pathway for nirmatrelvir in unchanged form. This reduces inter-individual variability and allows for a more predictable pharmacokinetic profile. It is worth noting that nirmatrelvir’s half-life is approximately 6 h with the “boosting” of ritonavir; thus, the twice-daily dosing is recommended to maintain the therapeutic concentrations. Without ritonavir, nirmatrelvir would be cleared too quickly [92,93,94,95].

Nirmatrevil shows moderate inhibition of OATP 1A2, 1B1, 1B3, and 2B1 only at high concentrations, suggesting limited transporter interaction potential on its own. However, nirmatrevil combined with ritonavir, as in Paxlovid™, significantly inhibits OATP transporters at clinically relevant concentrations, primarily due to ritonavir. Paxlovid™ inhibits ABC transporters (e.g., P-gp), highlighting ritonavir’s dominant role in transporter-mediated drug interaction [57,80].

Nirmatrelvir and ensitrelavir both target the SARS-CoV-2 M^pro^, acting upstream in the replication cycle by preventing polyprotein processing. Targeting M^pro^ is highly conserved; therefore, the two drugs are considered minimally affected by CES and transporters, unlike the nucleoside prodrugs. Another advantage of ensitrelavir is that it does not require ritonavir co-administered, avoiding ritonavir broadening CYP inhibition. While ritonavir boosts drug levels, CES-independent degradation can influence nirmatrelvir therapeutic success. Proper dosing and timing are important since nirmatrelvir may require early use to block protease before viral replication and worsening symptoms [95]. Moreover, molnupiravir and other options may become useful when the virus develops M^pro^ resistance, but potential drug resistance may evolve under subtherapeutic exposure due to suboptimal CES-mediated activation. Thus, a new generation of protease inhibitors, ensitrelavir, is currently under investigation for COVID-19 prevention, as the drug is ideal for patients with immune suppression. The relationship between the key enzymes in the context of immune-compromised patients remains a challenge. Therefore, we propose investigating the localization of the enzymes involved in SARS-CoV-2 infection across various cell lines representing different targeted sites of action.

## 6. Implications and Conclusions

While it is well established that COVID-19 can broadly suppress xenobiotic metabolism pathways, the impact of SARS-CoV-2 infection on CES1 and CES2 activity remains underexplored, and experimental validation in hepatocytes or in vivo models needs to be performed. This review highlights the clinical significance of CES1 and CES2 genetic polymorphisms, particularly concerning remdesivir and monupiravir. Given the role of kinases in the phosphorylation of the activation of remdesivir and possibly monupiravir, systematic profiling of kinase expression and activity across cell types could help refine predictions of antiviral efficacy. Yet, there is a lack of clinical trials for prodrugs requiring multi-step activation pathways, where hydrolysis is followed by phosphorylation. CES gene variants affect drug activation, thus, raising concerns about altered drug activation, distribution, and elimination, necessitating further consideration of their influence on transmembrane transport. While CES1 polymorphisms such as G143E can impair hydrolytic activation, additional genetic mechanisms of CES induced during SARS-CoV-2 infection may further compromise drug activation. Future studies should further explore and include the regulatory mechanisms affecting CES expression during SARS-CoV-2 infection, including transcriptional control by factors such as activating transcription factors (ATF). For example, ATF3 can increase hepatic CES1 and CES2 protein levels.

Cellular metabolizing enzymes and transporters significantly impact the fate of common antiviral medications used for SARS-CoV-2 infection. In conclusion, remdesivir and molnupiravir show a complex relationship between ACE2/TMPRSS2 expression at the entry sites and the intracellular availability of CES (activation), ENT (uptake), and CYP enzymes (metabolism or inactivation), suggesting that host genetic and cellular expression profiles may influence antiviral efficacy and toxicity. The connection may extend to nirmatrevir, where the coordinated activity of the enzymes also determines therapeutic outcomes. We provide a summary table for clinicians that bridges CES with drug-specific activation pathways and interactions in COVID-19 treatment, especially for complex treatment regimens (Table S1).

CES genotyping has been explored in other therapeutic areas (Table 2 and Table 3), where functional polymorphisms have been associated with altered drug response. We suggest developing CES-based diagnostics, including genotyping as a foundational tool or clinical guidelines supporting CES1/2 genotyping for antiviral therapy. The efficacy of antiviral drugs against SARS-CoV-2 is challenged not only by host-related metabolic variability (e.g., CES polymorphisms) but also by viral resistance, which is critical for long-term treatment strategies. The interplay of viral mutations and host enzymatic activation presents a significant challenge related to inefficient CES activation and patient-specific CES profiles, especially under COVID-19 conditions.

Targeted delivery strategies, particularly those focusing on CES2-rich intestinal tissues, offer an opportunity to maximize the bioavailability and optimize the activation and metabolism of prodrugs like molnupiravir or novel remdesivir analogs with desired dosage forms and routes of administration, less frequent dosing, and reduced toxicity. Furthermore, the development of precision antiviral therapies that correspond with patient genotypes and tissue-specific expression patterns can also be made possible by shifting the focus toward cell-type-specific drug activation by integrating local enzyme expression and regulation. This strongly supports the potential of personalized medicine in antiviral therapy.

## Figures and Tables

**Table 2:** List of genetic polymorphisms of CES1 affecting the metabolism of COVID-19 drug.

Variant		Prevalence	Drug Metabolism	Note
G143E	Amino acid glycine (Gly) at position 143 is replaced by glutamic acid (Glu)	Relatively low frequency of G143E heterozygotes [43,44,45,46,47,48]. G143E is rare in most populations but shows measurable frequencies in Europeans (3–4%) and Hispanics (1–2%)	Alters metabolism of enalapril, a CES1 substrate, leading to variations in drug efficacy and side effects [46,47]	Potentially influence remdesivir activation and efficacy, thus, requiring dose adjustment based on individual genetic profile for better efficacy or to avoid adverse reactions.
rs2244613 (c.1165-33C>A)	Single-nucleotide polymorphism (SNP) in the CES1 gene that involves a change of C to A at position 1165-33.	Low frequency of rs2244613 heterozygotes and homozygotes [47,49,50,51] Approximately 20–25% of Europeans, 15–20% of East Asians, and 10–15% of Africans have rs2244613.	The frequency of rs2244613 suggests rs2244613-relevant dosing of enalapril [47]. Affects the conversion of capecitabine to 5-fluorouracil, increasing the risk of capecitabine-induced adverse events (hand-foot syndrome) [49] May be associated with CYP enzyme activity ^1^ [51,52] Connected with the ABCB1 gene ^2^ [50]	
c.662A>G	SNP in the CES1 gene, which involves a change of A to G at position 662.	Relatively common, with the homozygous for c.662AA being the most prevalent [54].	Individuals with the c.662A>G genotype may have altered CES1 activity, which can affect the efficacy and safety of oseltamivir [54].	
rs8192935	SNP variants in the CES1 gene	Relatively common	Connected with the ABCB1 gene ^2^ [50]	
rs4784563		Fairly common	Connected with the ABCB1 gene ^2^ [55] Help optimize dabigatran dose ^3^ [55]	
rs4580160		Fairly common	Help optimize dabigatran dose ^3^ [55]	
rs4122238		Fairly common, but rs4122238 can exhibit different minor allele frequency values depending on the specific population studied [55,56]	Showed impacts on how individuals respond to methylphenidate [56] and dabigatran ^3^ [55]	

^1^ A gene that affects CYP can potentially influence the metabolism of nirmatrelvir, which is metabolized primarily by CYP enzymes, including CYP3A4 and CYP3A5. ^2^ The ABCB1 gene encodes the P-gp, a drug efflux pump, which is involved in the transport and metabolism of ritonavir [57]. ^3^ Dabigatran is administered orally as a prodrug. Upon entering the body, dabigatran is converted to active metabolite by CES1 and 2. Dabigatran is primarily metabolized by P-gp and CYP enzymes [58].

**Table 3:** List of genetic polymorphisms of CES2 affecting the metabolism of COVID-19 drug.

Variant		Prevalence	Drug Metabolism	Note
rs2241409 (c.1613-108G>A)	SNP in the CES2 gene, which involves a change of G to A at position c.1613-108.	Relatively low frequency of rs2241409 homozygotes. rs2241409 is more frequent in Asian populations.	Heterozygotes ^1^ might have an intermediate response to capecitabine, possibly resulting in variable efficacy or toxicity [49]	Potentially influence remdesivir and molnupiravir activation and efficacy
rs11075646 (c.-806C>G, previously referred to as c.-823C>G)	SNP in the CES2 gene, which involves a change of C to G in the 5′-untranslated region.	Very low frequency	Associated with capecitabine-induced risk of hand-foot syndrome and better response and time to tumor progression, may play a role in the metabolism of capecitabine [65]. The combined influence of rs2241409 and rs11075646 could provide further insight into individual responses to treatment with capecitabine [66].	
∆458-473	Deletion of residues 458–473 in the CES2 enzyme.		Showed fully destroyed hydrolytic activity towards irinotecan [67].	
A139T and F458V	Two specific nonsynonymous SNPs in CES2		Associated with decreased hydrolysis of aspirin in vitro [68]	
rs72547531 (c.100C>T, R34W)	SNP in the CES2 gene, which involves a change of C to T.		Result in decreased esterase activity towards irinotecan as well as two typical carboxylesterase substrates (p-nitrophenol acetate and 4-methylumbelliferyl acetate) [69]	
rs72547532 (c.424G>A, V142M)	SNP in the CES2 gene, which involves a change of G to A.			
IVS8-2A>G	Deletion of 32 base pairs in exon 9 at the splice acceptor site in intron 8, resulting in aberrant splicing, producing truncated CES2 proteins		The aberrant CES2 proteins exhibit impaired activity, significantly reducing CES2 hydrolytic function, and affecting drug metabolism [69]	

^1^ Heterozygous carriers (G>A) may experience a moderate effect on metabolism. Homozygous carriers (A>A) could either experience faster or slower metabolism, depending on whether the variant leads to reduced enzyme activity or altered gene expression, thus, influencing clinical outcomes.

## Data Availability

The authors confirm that the data supporting this study are within the article.

## Supplementary Materials

The following supporting information can be downloaded at: https://www.mdpi.com/article/10.3390/pharmaceutics17070832/s1, Table S1: CES-mediated activation of antiviral drugs and potential drug-drug interactions.

## Abbreviations

The following abbreviations are used in this manuscript:

SARS-CoV-2	severe acute respiratory syndrome coronavirus 2
ACE2	angiotensin-converting enzyme 2
M^pro^	main protease
3CL^pro^	3-chymotrypsin-like proteases
RdRp	RNA-dependent RNA polymerase
CES	carboxylesterases
CYP	cytochrome P450
S	spike
RBD	receptor-biding domain
TMPRSS2	transmembrane protease, serine 2
FCS	furin cleavage site
GI	gastrointestinal
NHC	β-D-*N*^4^-hydroxycytidine
RTP	remdesivir triphosphate
EUA	Emergency Use Authorization
ORF	open reading frames
nsps	non-structure proteins
P-gp	P-glycoprotein
SNP	single nucleotide polymorphism
TAF	tenofovir alafenamide
TFV	tenofovir
PBMC	peripheral blood mononuclear cells
SLC	solute carrier
OATP	organic anion transporting polypeptide
ENT	equilibrative nucleoside transporter
MDCK	Madin-Darby canine kidney
CNT	concentrative nucleoside transporter
