# Supplementary material for: Carboxylesterase Factors Influencing the Therapeutic Activity of Common Antiviral Medications Used for SARS-CoV-2 Infection
Source: Pharmaceutics. 2025 Jun 26;17(7):832. doi: 10.3390/pharmaceutics17070832 (PMC12298093; doi:10.3390/pharmaceutics17070832)
Supplement: Supplementary file 1 [file pharmaceutics-17-00832-s001.zip › pharmaceutics-3603042-supplementary.pdf]

Review

Carboxylesterase Factors Influencing the Therapeutic Activity of Common Antiviral Medications Used for SARS-CoV-2 Infection

Yue Shen †, William Eades, Linh Dinh *,† and Bingfang Yan *

Division of Pharmaceutical Sciences, James L. Winkle College of Pharmacy, University of Cincinnati, Cincinnati, OH 45229, USA
* Correspondence: dinhlk@ucmail.uc.edu (L.D.); yanbg@uc.edu (B.Y.); Tel.: +1-513-558-6297 (B.Y.)
† These authors contributed equally to this work.

Table S1. CES-mediated activation of antiviral drugs and potential drug-drug interactions

Antivirals	Activation Pathway	Broader Activation Pathway Involved Other Enzymes/Transporters		Clinical Implications and Potential Drug-Drug Interactions
Veklury® (Remdesivir)	Hydrolyzed by CES1 → active nucleotide analog (phosphorylation/dephosphorylation)	• hydrolyzed by Cathepsin A<br>• oxidized by CYP3A4, CYP2B6, CYP2C8, CYP2D6<br>• transported by ENT and CNT<br>• inhibits CES2 irreversibly at low concentrations<br>• kinases and phosphatases can active/inactive drug by phosphorylating/dephosphorylating		• CYP interactions, particularly interactions with CYP3A4 inhibitors/inducers<br>• affect CES2 substrates<br>• caution with co-administering drugs that affect kinase/ phosphatase activity
LAGEVRIO™ (Molnupiravir)	Hydrolyzed by CES2 → active nucleotide analog (phosphorylation/dephosphorylation)	• oxidized by CYP but minor CYP involvement.<br>• transported by ENT and CNT but less dependent on transporters compared to remdesivir.<br>• kinases and phosphatases can active/inactive drug by phosphorylating/dephosphorylating		• lower CYP interaction risks compared to remdesivir<br>• activation impaired by CES2 inhibitors<br>• caution with co-administering drugs that affect kinase/ phosphatase activity
Paxlovid™ (Nirmatrelvir/Ritonavir)	-	nirmatrelvir	• oxidized by CYP, particularly CYP3A4<br>• inhibits OATP 1A2, 1B1, 1B3, and 2B1 at high concentrations	• High potential of CYP interaction, particularly CYP3A subtrates
		ritonavir	• inhibits CYP and P-gp	
